# The Impact of Whey Protein Supplementation on Sarcopenia Progression among the Elderly: A Systematic Review and Meta-Analysis

**DOI:** 10.3390/nu15092039

**Published:** 2023-04-23

**Authors:** Magdalena Sylwia Kamińska, Kamila Rachubińska, Szymon Grochans, Karolina Skonieczna-Żydecka, Anna Maria Cybulska, Elżbieta Grochans, Beata Karakiewicz

**Affiliations:** 1 Subdepartment of Long-Term Care and Palliative Medicine, Department of Social Medicine, Faculty of Health Sciences, Pomeranian Medical University in Szczecin, 48 Żołnierska, 71-210 Szczecin, Poland; magdalena.kaminska@pum.edu.pl; 2 Department of Nursing, Faculty of Health Sciences, Pomeranian Medical University in Szczecin, 48 Żołnierska, 71-210 Szczecin, Poland; 3 Department of Clinical Nursing, Faculty of Health Sciences, Pomeranian Medical University in Szczecin, 48 Żołnierska, 71-210 Szczecin, Poland; 4 Department of Biochemical Science, Faculty of Health Sciences, Pomeranian Medical University in Szczecin, 24 Broniewskiego, 71-460 Szczecin, Poland; 5 Subdepartment of Social Medicine and Public Health, Department of Social Medicine, Faculty of Health Sciences, Pomeranian Medical University in Szczecin, 48 Żołnierska, 71-210 Szczecin, Poland

**Keywords:** sarcopenia, older adults, whey protein

## Abstract

We conducted a systematic literature review and meta-analysis to investigate the role of whey protein supplementation in the functioning of the elderly with sarcopenia. The aim was to investigate the available scientific evidence and determine the best recommendations with respect to whey protein supplementation in sarcopenic patients. Methods: Databases, including CINAHL, Embase PubMed, and Web of Science, were searched from database inception until 31 December 2022 for randomised controlled trials (RCTs) comparing the efficacy of whey protein supplementation in the elderly with sarcopenia. Data on study design, risk of bias, patient, illness, and treatment characteristics from each study were independently extracted in accordance with the Preferred Reporting Items for Systematic Reviews and Meta-Analyses (PRISMA). The tool “assessing risk of bias” from the Cochrane Handbook was used to evaluate the quality of the included papers. Results: The search identified 629 records; 590 articles were excluded as duplicates or after evaluation at the title or abstract level. Out of 39 full-text articles that were reviewed, 29 were excluded for not fulfilling the inclusion criteria. There is some evidence that whey protein supplementation combined with age-appropriate physical exercise might improve muscle mass and lower limb function in the elderly with sarcopenia. The present meta-analysis demonstrated overall that whey supplementation does not improve any of the tested sarcopenia-linked parameters. However, we found that study duration (weeks) and age significantly affect the handgrip strength rate and the chair and stand test rate, respectively, so consideration should be given to oral supplementation combined with the age of participants and an appropriate physical activity as a form of sarcopenia prevention in the high-risk group.

## 1. Introduction

The World Health Organisation (WHO) declared 2020–2030 to be the Decade of Healthy Ageing. It is estimated that by 2050, one in five people will be over 60 years of age [1]. Given the ageing of populations, it is essential to ensure “healthy ageing” defined as “a lifelong process optimizing opportunities for improving and preserving health and physical, social and mental wellness, independence, quality of life and improving positive changes throughout life” [2].

Sarcopenia is a condition that significantly affects ageing. The International Sarcopenia Consensus Conference Working Group defines sarcopenia as “a muscle disease (muscle failure) rooted in adverse muscle changes that accrue across lifetime” [3]. The term ‘sarcopenia’ was originally defined by Rosenberg in 1989 as loss of muscle mass using the Greek “sarx” for flesh and “penia” for loss [4]. It was only after some time that sarcopenia was listed as a muscle disease in the International Classification of Diseases (ICD-10:M62) [5]. Sarcopenia is related to advanced ageing and begins before the age of 60 [3,6]. It is worth noting that a decrease in muscle mass begins as early as after the age of 40 [3]. The prevalence of sarcopenia in people over 60 and 80 years of age is estimated at 5–13% and 11–50%, respectively [7,8]. According to the latest consensus, the key features of sarcopenia are as follows:

(1) low muscle strength (tested with handgrip strength and chair stand tests);
(2) low muscle quantity or quality (confirmed with dual-energy X-ray absorptiometry (DEXA), computed tomography (CT), magnetic resonance imaging (MRI), etc.);
(3) diminished physical performance (assessed with gait speed tests, 400-metre walking tests, etc.) [3].

There is scientific evidence indicating the key importance of protein consumption in the prevention of sarcopenia and maintaining lean body mass [9,10,11,12]. Unfortunately, ageing has a significant effect on the nutritional requirements of the elderly as well as protein metabolism [13]. Seniors are at increased risk of protein-energy malnutrition due to loss of appetite and potential problems with chewing or social isolation [14]. Proteins increase the feeling of satiety; therefore, the elderly are less likely to meet the protein demand as a result of the said feeling of satiety and skipping meals. Protein is a key macronutrient for maintaining the proper functioning of the body [15]. As the elderly are often unable to meet the daily protein demand as recommended by nutritionists (from 1.0 to 1.2 g/kg of body weight daily) [16], protein supplementation is vital [13]. There are different oral protein supplements available, mainly soybean- or cow’s milk-based. The latter includes whey protein, which is considered the best source of oral protein supplementation. Whey protein contains amino acids and bioactive peptides that reduce postprandial glucose fluctuations via insulin-dependent and insulin-independent mechanisms [17]. Additionally, the main advantages of whey protein are its high leucine content, high digestibility, and availability of amino acids [18,19]. Therefore, in order to reduce the risk of developing sarcopenia in this risk group, it is crucial to provide a balanced, nutrient-rich source of protein that maximises the mass and strength of muscles.

Given the important issue of population ageing and the dietary problems of the elderly, there is an increased risk of the occurrence of sarcopenia. We conducted a systematic literature review and meta-analysis to investigate the role of whey protein supplementation on several aspects of sarcopenia progression in the elderly. The aim was to investigate the available scientific evidence and determine the best recommendations with respect to whey protein supplementation in sarcopenic patients.

The biggest novelty of our systematic review with meta-analysis, as compared to other meta-analyses testing the efficacy of whey protein as a prevention measure towards sarcopenia, is meta-regression analysis with the following covariates: study duration (weeks), percentage of males in the population, age of the participants, dose of the active product, percentage of the analysed participants, changes in appendicular muscle mass rate, handgrip strength, chair and stand test rate, SPPB, and weight by protein intake.

## 2. Materials and Methods

### 2.1. Search Strategy and Inclusion Criteria

At least two independent authors (M.K., K.R.) searched CINAHL, Embase PubMed, and Web of Science from database inception until 31 December 2022 with language restriction (only English, Polish, and German) for randomised controlled trials (RCTs) comparing the efficacy of whey protein supplementation in the elderly with sarcopenia.

The following search terms were used in PubMed/Web of Science/Cinahl: ((sarcopenia) AND (older people OR older adults OR aged OR aged patient OR aged people OR aged person OR aged subject OR elderly OR elderly patient OR elderly people OR elderly person OR elderly subject OR senior citizen OR senium) AND (whey protein OR milk whey protein OR resource whey protein OR whey protein OR whey proteins) AND (placebo OR placebos OR physical activity OR activity, physical OR no intervention) AND (randomised controlled trial OR controlled trial, randomised OR randomised controlled study OR randomised controlled study OR randomised controlled trial OR trial, randomised controlled)).

In Embase, the following search string was used: (‘sarcopenic obesity’/exp OR ‘sarcopenic obesity’ OR ‘sarcopenia’/exp) AND (‘older people’/exp OR ‘older adults’/exp OR ‘aged’/exp OR ‘aged’ OR ‘aged patient’ OR ‘aged people’ OR ‘aged person’ OR ‘aged subject’ OR ‘elderly’ OR ‘elderly patient’ OR ‘elderly people’ OR ‘elderly person’ OR ‘elderly subject’ OR ‘senior citizen’ OR ‘senium’) AND (‘whey protein’/exp OR ‘beneprotein’ OR ‘milk whey protein’ OR ‘resource whey protein’ OR ‘whey protein’ OR ‘whey proteins’) AND (‘placebo’/exp OR ‘placebo’ OR ‘placebo gel’ OR ‘placebos’ OR ‘physical activity’/exp OR ‘activity, physical’ OR ‘physical activity’ OR ‘no intervention’) AND (‘randomised controlled trial’/exp OR ‘controlled trial, randomised’ OR ‘randomised controlled study’ OR ‘randomised controlled trial’ OR ‘randomised controlled study’ OR ‘randomised controlled trial’ OR ‘trial, randomised controlled’).

The electronic search was supplemented by a manual review of reference lists from eligible publications and relevant reviews. Inclusion criteria were as follows:

(1) Human studies (studies in adults >60 years old);
(2) Patients with diagnosed sarcopenia;
(3) Languages: Polish, English, and German;
(4) Randomised placebo-controlled clinical trial;
(5) Intervention: whey protein supplementation compared to placebo/control group.

We excluded the following studies:

(1) In animals;
(2) Comprising non-sarcopenic patients;
(3) Studies related to interventions other than whey protein (protein supplementation, general nutritional supplementation, and leucine supplementation).

### 2.2. Data Abstraction

Data on study design, risk of bias [20], patient, illness, and treatment characteristics from each study were independently extracted in accordance with the Preferred Reporting Items for Systematic Reviews and Meta-Analyses (PRISMA) standard by two independent investigators (M.S.K., K.R.). Whenever data was missing for the review, authors were contacted for additional information twice, at least two weeks apart. Inconsistencies were resolved by consensus with a senior investigator (K.S.-Ż.).

### 2.3. Outcomes

The primary outcome was data on physical function after the whey protein supplementation, i.e., muscle performance/physical performance measures/risk for falls measured with the advent of appropriate techniques/methods. 

The secondary outcomes included nutritional assessment and biochemical parameters after the intervention.

### 2.4. Data Synthesis and Statistical Analysis

We conducted a random effects [21] meta-analysis of outcomes for which ≥ 3 studies contributed data using Comprehensive Meta-Analysis V3 (http://www.meta-analysis.com). We explored study heterogeneity using the chi-square test of homogeneity, with *p* < 0.05 indicating significant heterogeneity. All analyses were two-tailed with an alpha = 0.05.

Group differences in continuous outcomes were analysed as the pooled standardised mean difference (SMD) in either endpoint scores (preferred) or change from baseline to endpoint using observed cases (OC). Categorical outcomes were analysed by calculating the pooled risk ratio (RR) using OC data. 

We conducted subgroup and exploratory maximum likelihood random-effects meta-regression analyses of the co-primary and secondary outcomes. Meta-regression variables included: (1) study duration (weeks), (2) percentage of males in the population, (3) age of the participants (mean), (4) dose of the active product, and (5) percentage of the analysed participants. Finally, we inspected funnel plots and used Egger’s regression test [22] and Duval and Tweedie’s trim and fill method [23] to quantify whether publication bias could have influenced the results.

### 2.5. Risk of Bias

Two authors (M.K. and K.R.) independently assessed the risk of bias using the Cochrane Collaboration tool for assessing the risk of bias [20]. When a discrepancy occurred, a third author (K.S.-Ż.) was involved. Arbitrarily, we made the assumption that the quality of a study was reported as high when there were more than three low-risk bias assessments.

## 3. Results

### 3.1. Search Results

**Figure 1:**
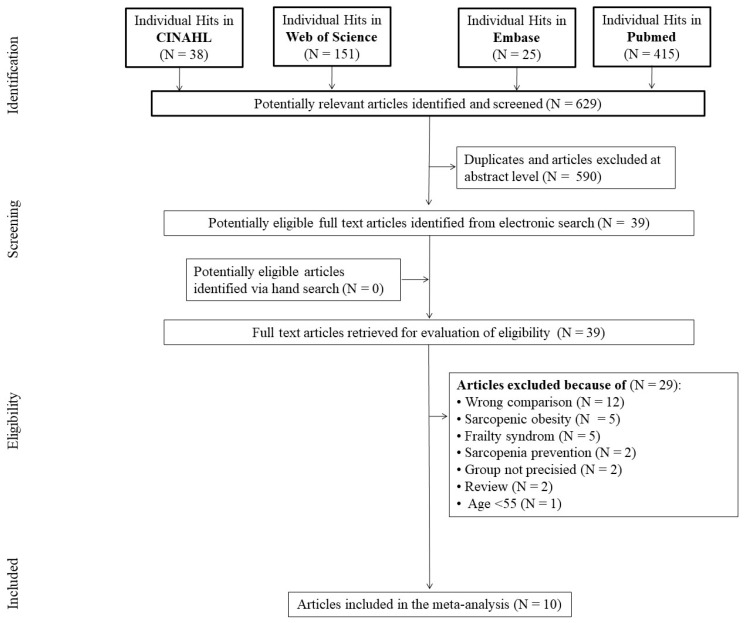
Flow diagram of included and excluded studies.

The search identified 629 records, of which 590 articles were excluded as duplicates or after evaluation at the title or abstract level. Out of 39 full-text articles that were reviewed, 29 were excluded for not fulfilling the inclusion criteria. Primary reasons for exclusion were: wrong comparison [protein supplementation, general nutritional supplementation, and leucine supplementation] (*n* = 12), sarcopenic obesity (*n* = 5), frailty syndrome (*n* = 5), sarcopenia prevention (*n* = 2), group not precise (*n* = 2), review (*n* = 2), and age < 55 years (*n* = 1), yielding ten studies that were included in the meta-analysis (Figure 1).

### 3.2. Characteristics of the Included Studies

The present meta-analysis included ten studies published in 2015–2022 [24,25,26,27,28,29,30,31,32,33,34]. The studies were conducted as a multicentre study in Europe (Belgium, Germany, Ireland, Italy, Sweden, and the United Kingdom) [24,25,26,29,30,31,32,33] (*n* = 8), the Near East (*n* = 1) [28], and East Asia (*n* = 1) [27] (Table 1).

Three studies were non-industry funded [26,27,28]. Six studies were industry funded [24,25,29,30,31,33]. In one study, no sources of funding were indicated [30]. (Table 1).

There were 2498 patients included, with a predominance of females (*n* = 1373, 54.96%). The size of the study groups ranged from 60 to 380 people. The overall mean age of the participants for the ten studies was between 74.03 and 83.77 years. Mostly, all the included studies employed an intervention period of 13 weeks [24,25,28,29,30,33]. One study had an intervention period of 52 weeks [26]. Other studies lasted 26 [27] or 12 weeks [31], with one study having a short intervention period of 4–8 weeks [32]. All the studies had interventions with either whey protein or placebo [24,25,26,27,28,29,30,31,32,33]. Mostly, all the included studies used products from WP-MND (FortiFit, Nutricia N.V., the Netherlands [24,25,29,30,33] (Table 1).

There were six studies providing data on the same study group subjected to the same intervention [24,25,28,29,30,33]. Indeed, they were the sub-studies of the PROVIDE trial corresponding to the references we cited. That is why in the present paper, data was used for calculations only once (from one paper only, which constituted the main trial) [24].

### 3.3. Characteristics of Sarcopenia

Table 2 shows a summary of the main characteristics and measurements of sarcopenia in the analysed studies. Ten studies enrolled community-dwelling elderly individuals with sarcopenia [24,25,26,27,28,29,30,31,32,33]. Almost all of the studies included in the assessment of sarcopenia with recommended tools (EWGSOP) [24,25,26,27,28,30,31,32,33] are indicated in Table 2.

### 3.4. Muscle Mass, Muscle Strength, and Physical Performance

There were six articles evaluating body composition using Dual-energy X-ray absorptiometry (DXA) and five articles evaluating body composition using Bioelectrical Impedance Analysis (BIA). Five articles evaluated Body Mass Index (BMI), and six—Mini Nutritional Assessment (MNA), of which two evaluated Mini Nutritional Assessments—Short Form (MNA-SF). There were seven articles evaluating muscle strength with handgrip dynamometry. Physical performance assessment was conducted using SPPB, chair stand time, balance test, and gait speed.

### 3.5. Characteristics of Interventions

The mean study duration was 17.4 ± 13.1 weeks (range = 4–26 weeks). A daily dose of active product in most of the articles was 40 g [26,28,30,32,33], in two of them—20 g [29], 22 g [31], 23 g [27], 42 g [25], and 80 g [24]. The study by Björkman et al. [26] had three arms and the following interventions: (1) control with no supplementation, (2) isocaloric placebo, and (3) whey protein. All groups were given instructions on home-based exercise, dietary protein, and vitamin D intake.

### 3.6. Quality Assessment

The Risk of Bias Assessment

The risk of bias was assessed by the Cochrane Handbook for the Systematic Review of Interventions in the selected studies [34]. The mean number of low-risk-of-bias assessments in all studies included in the meta-analysis was 4.3 (median = 5). The analysis of the risk of bias assessment demonstrated that only one study showed low quality [28]. Details of the risk of bias evaluation are given in Table 3.

### 3.7. Meta-Analysis

#### Outcome Measures

The Effect of Whey Protein Intake on Muscle Mass and Handgrip Strength

**Figure 2:**
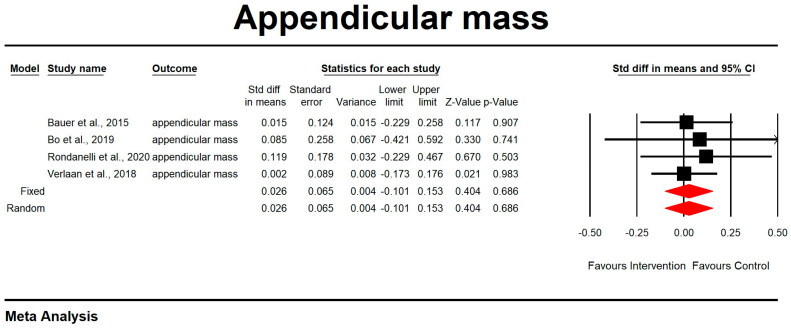
The effect size for appendicular muscle mass rate when using whey protein intake vs. placebo. Q = 0.409, df (Q) = 3, *p* = 0.938, and I squared = 0.000. Black squares depict each study effect size; red diamonds represent merged effect sizes calculated in mixed and random models [24,27,32,33].

**Figure 3:**
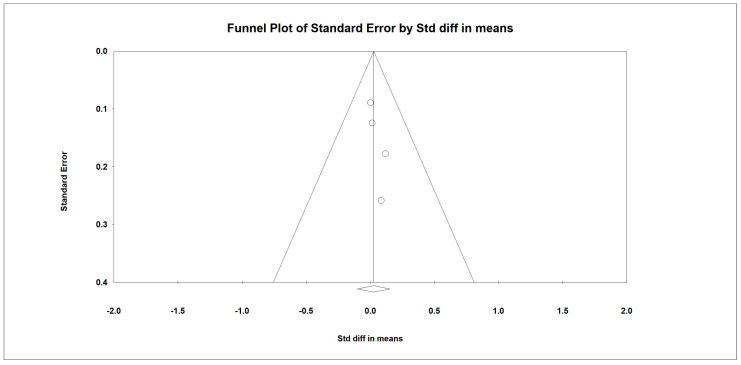
Funnel plot for appendicular muscle mass rate in the present meta-analysis.

Regarding the effect of protein intake on appendicular muscle mass, the data showed that whey protein intake did not significantly affect appendicular muscle mass. The results are presented in Figure 2 (*p* = 0.686; Z = 0.404; CI 95%: −0.101–0.153). Egger’s test did not indicate publication bias, as shown in Figure 3. (*t* value = 2.16554, *p* = 0.08136). No heterogeneity was detected.

**Figure 4:**
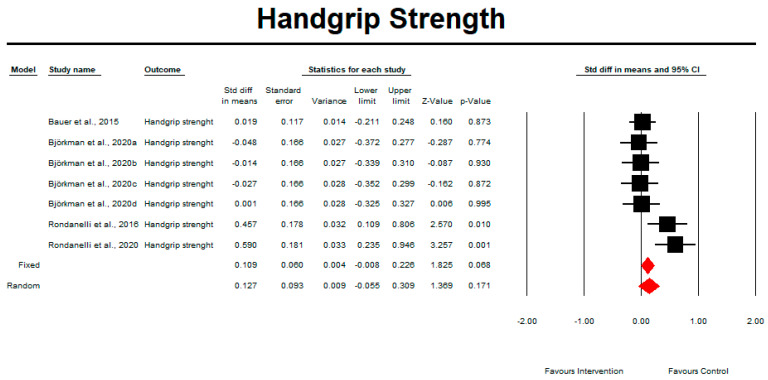
The effect size for handgrip strength when using whey protein intake vs. placebo. Q = 14.023, df (Q) = 6, *p* = 0.029, and I squared = 57.212. Black squares depict each study effect size; red diamonds represent merged effect sizes calculated in mixed and random models [24,26,31,32].

**Figure 5:**
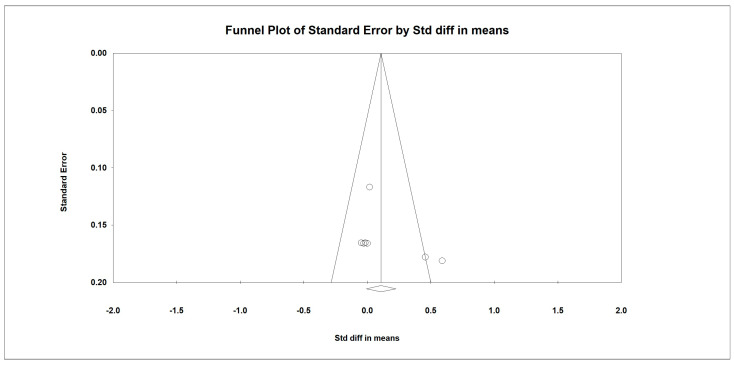
Funnel plot for handgrip strength rate in the present meta-analysis.

Regarding the effect of whey protein intake on handgrip strength, the data showed no difference compared with the control intervention, as shown in Figure 4 (*p* = 0.171; Z = 1.369; CI 95%: −0.055–0.309). Egger’s test did not indicate publication bias, as shown in Figure 5 (*t* value = 1.14660, *p* = 0.15172).

### 3.8. The Effect of Protein Intake on Physical Performance

Chair and Stand Test

**Figure 6:**
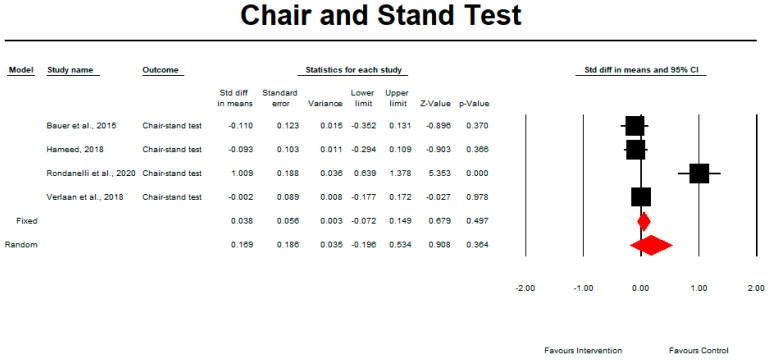
The effect size for chair and stand test rate when using whey protein intake vs. placebo. Q = 29.810, df (Q) = 3, *p* = 0.000, and I squared = 89.936. Black squares depict each study effect size; red diamonds represent merged effect sizes calculated in mixed and random models [24,28,32,33].

**Figure 7:**
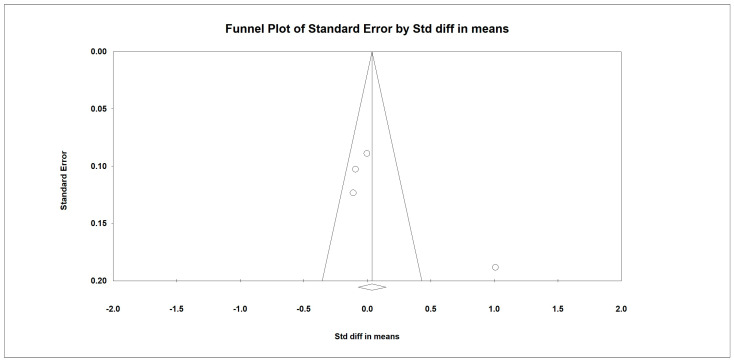
Funnel plot for chair and stand test rate in the present meta-analysis.

Regarding the effect of whey protein intake on physical performance, the data showed that there were no changes in the chair and stand test, as shown in Figure 6 (*p* = 0.364; Z = 0.908; CI 95%: −0.196–0.534). Egger’s test did not indicate publication bias, as shown in Figure 7 (*t* value = 2.00908, *p* = 0.09114). When we excluded an outlier study by Rondanelli et al. (2020), the results remained unchanged (*p* = 0.864; Z = 0.171; CI 95%: −0.125–0.149).

### 3.9. SPPB

**Figure 8:**
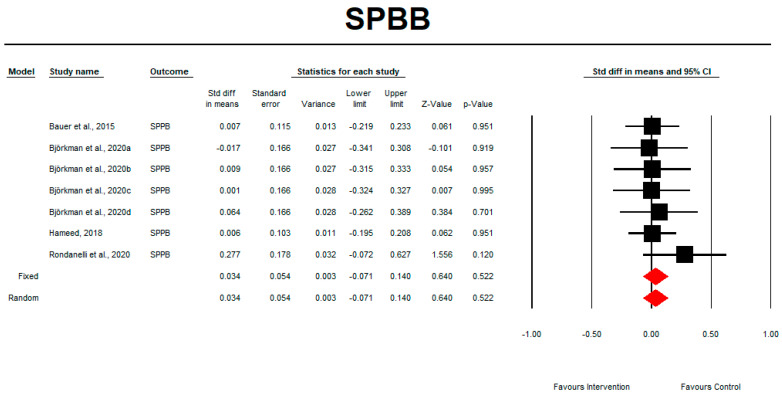
The effect size for SPPB rate when using whey protein intake vs. placebo. Q = 2.178, df (Q) = 6, *p* = 0.903, and I squared = 0.000. Black squares depict each study effect size; red diamonds represent merged effect sizes calculated in mixed and random models [24,26,28,32].

**Figure 9:**
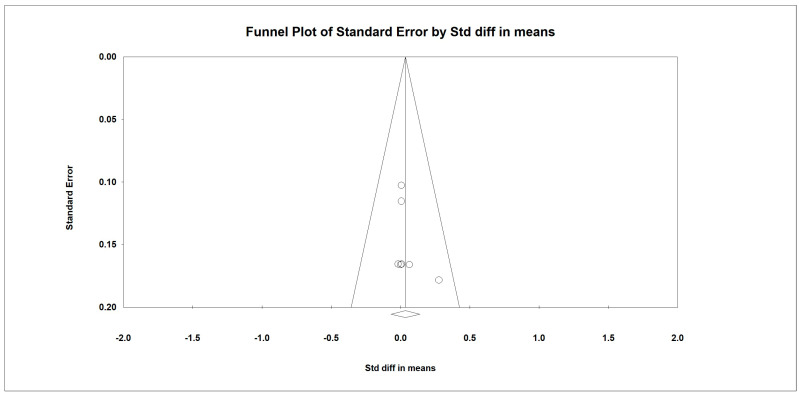
Funnel plot for SPPB rate in the present meta-analysis.

Regarding the effect of whey protein intake on physical performance, the data showed that whey protein intake did not significantly affect SPPB, as shown in Figure 8 (*p* = 0.552; Z = 0.640; CI 95%: −0.071–0.140). Egger’s test did not indicate publication bias, as shown in Figure 9 (*t* value = 1.09617, *p* = 0.16149).

### 3.10. Weight

**Figure 10:**
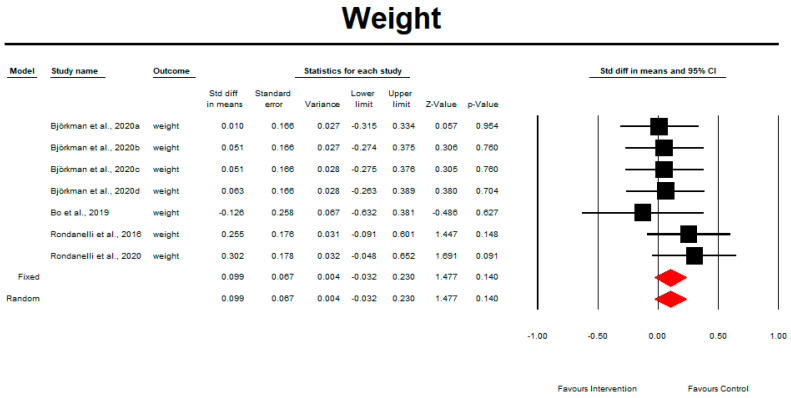
The effect size for weight rate when using whey protein intake vs. placebo. Q = 3.341, df (Q) = 6, *p* = 0.765, and I squared = 0.000. Black squares depict each study effect size; red diamonds represent merged effect sizes calculated in mixed and random models [26,27,31,32].

**Figure 11:**
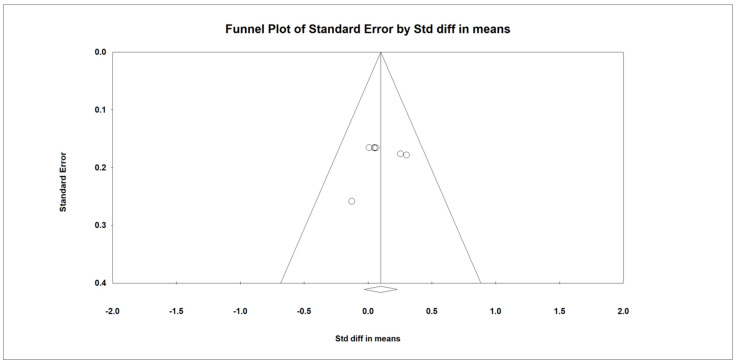
Funnel plot for weight in the present meta-analysis.

Regarding the effect of whey protein intake on physical performance, the data showed that there were no changes in weight, as shown in Figure 10 (*p* = 0.140; Z = 1.477; CI 95%: −0.032–0.230). Egger’s test did not indicate publication bias, as shown in Figure 11. *(t* value = 0.65839, *p* = 0.26970).

### 3.11. The Effect of Whey Protein Intake on MNA Results

**Figure 12:**
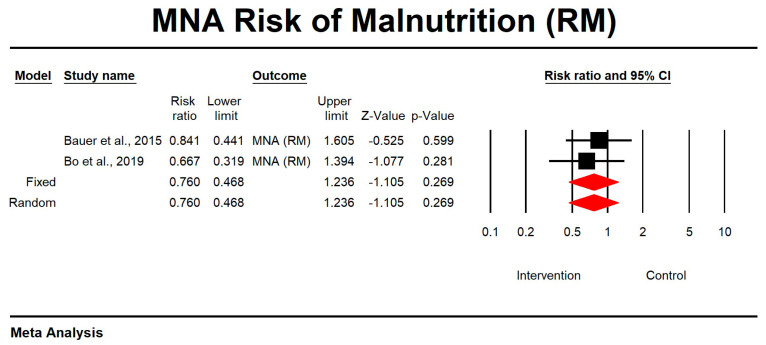
The effect size for risk of malnutrition according to MNA when using whey protein intake vs. placebo. Q = 0.215, df (Q) = 1, *p* = 0.643, and I squared = 0.000. Black squares depict each study effect size; red diamonds represent merged effect sizes calculated in mixed and random models [24,27].

Regarding the effect of whey protein intake on the risk of malnutrition according to MNA, the data showed that there were no changes in the risk of malnutrition, as shown in Figure 12 (*p* = 0.269; Z = −1.105; CI 95%: 0.468–1.236).

**Figure 13:**
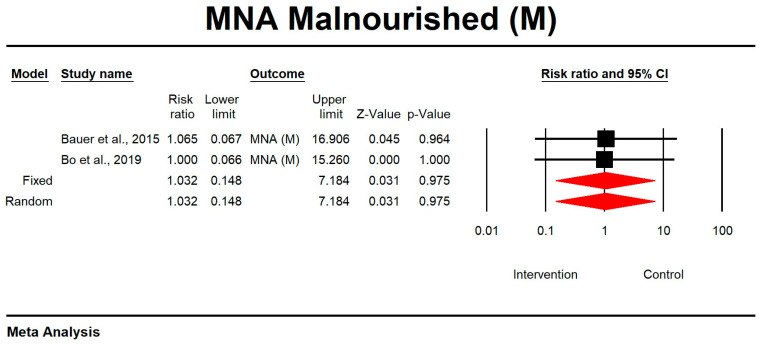
The effect size for malnourishment according to MNA when using whey protein intake vs. placebo. Q = 0.001, df (Q) = 1, *p* = 0.975, and I squared = 0.000. Black squares depict each study effect size; red diamonds represent merged effect sizes calculated in mixed and random models [24,27].

Regarding the effect of whey protein intake on malnourishment according to MNA, the data showed that there were no changes in the risk of malnutrition, as shown in Figure 13 (*p* = 0.975; Z = 0.031; CI 95%: 0.148–7.184).

**Figure 14:**
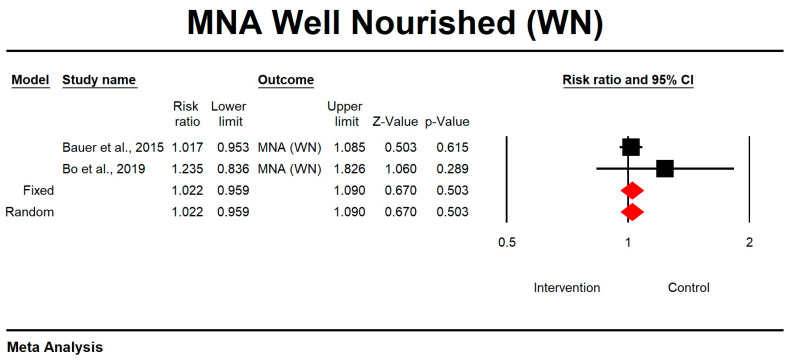
The effect size for well nourishment according to MNA when using whey protein intake vs. placebo. Q = 0.927, df (Q) = 1, *p* = 0.336, and I squared = 0.000. Black squares depict each study effect size; red diamonds represent merged effect sizes calculated in mixed and random models [24,27].

Regarding the effect of whey protein intake on well nourishment according to MNA, the data showed that there were no changes in the risk of malnutrition, as shown in Figure 14 (*p* = 0.503; Z = 0.670; CI 95%: 0.959–1.090).

### 3.12. Meta-Regression Analysis

In order to see whether there are some parameters affecting the observed effect sizes, we performed meta-regression analyses. The meta-regression analysis showed that, along with the increase in study duration, the effect on handgrip strength was smaller. However, one should remember that the coefficient is extremely low, and the majority of studies in this aspect were performed longer (around 13 weeks) than shorter (one study, 6 weeks). Additionally, a correlation was found between the chair and stand test results and study duration, age, and percentage of data analysed. Collectively, we found that the shorter the study duration, the older the participants, the smaller the study size, and the higher the effect size.

A meta-regression using a random-effects model revealed that the study duration (weeks) (Q = 5.01, df = 1, *p* = 0.0252 with coefficient = −0.0083, SE = 0.0037, Z = −2.24) significantly affected the handgrip strength rate (Table 4). A meta-regression using a random-effects model revealed that study duration (Q = 29.12, df = 1, *p* = 0.0000 with coefficient = −0.1522, SE = 0.0282, Z = −5.40), age of the participants (Q = 29.20, df = 1, *p* = 0.0000 with coefficient = 0.2822, SE = 0.0522, Z = 5.40), and percentage of analysed participants (Q = 29.12, df = 1, *p* = 0.0000 with coefficient = −0.0042, SE = 0.0008, Z = −5.40) significantly affect the chair and stand test rate (Table 4). No other statistically significant observations were made in a meta-regression analysis.

## 4. Discussion

So far, no effective method of preventing sarcopenia has been found. The issue is investigated by several groups: the European Working Group on Sarcopenia in Older People (EWGSOP), the International Working Group of Sarcopenia (IWGS), the Society of Sarcopenia, Cachexia, and Wasting Disorders (SCWD), and the Asian Working Group for Sarcopenia (AWGS). These working groups, as well as the authors of numerous studies, stress the role of proper nutrition and supplementation combined with appropriately dosed physical activity [35,36]. Hence, we conducted a systematic literature review and meta-analysis to investigate the role of whey protein supplementation and other interventions (e.g., physical exercise) in the functioning of elderly sarcopenic patients. The aim of this study was to investigate the available scientific evidence and identify the best recommendations with respect to whey protein supplementation for sarcopenic patients.

### 4.1. Main Findings

The main aspect taken into account in the present meta-analysis was the effectiveness of protein intake compared with other therapeutic interventions (including physical performance) used in sarcopenia treatment. Ten studies were selected for meta-analysis [24,25,26,27,28,29,30,31,32,33] because they presented the use of whey protein intake in comparison with alternative interventions.

It is interesting that in the present meta-analysis, strength was not affected by whey supplementation, but muscle mass did appear to generally improve. As pointed out by Esmarck et al. [37], factors such as the composition and quantity of protein supplementation and the consumption timing in relation to the resistance exercise training used have a significant impact on the improvement of muscle mass and function and a beneficial effect on muscle hypertrophy. While Park et al. [38] suggest that “the composition and timing of protein intake are more important than the total amount”.

Protein supplementation has no significant effect on improving the selected parameters of sarcopenia. The results are interesting as, until recently, whey protein intake has been the proposed standard in the treatment of sarcopenia in clinical practise. Early intervention with the rapid introduction of physiotherapy and whey supplementation may stop the progression of the disease and even reduce its negative effects.

### 4.2. Differences between Ours and Other Published Studies

The literature on the efficacy of whey protein in older adults as prevention against sarcopenia varies. Admittedly, in the systematic review and meta-analysis by Tu et al., which was conducted to explore the effect of protein intake on the prevention and improvement of sarcopenia, the authors included 12 articles and 872 participants that met the eligibility criteria in their review [39]. Their studies show that there were no significant changes in skeletal muscle mass with protein intake, and no difference in hand grip strength was observed with protein intake compared with control conditions. There were also no changes in the chair rise test or in SPPB. Moreover, protein intake did not have significant effects on the 4 m gait speed [39]. All the studies had interventions with either whey protein or a placebo. Mostly, the types of protein supplements included protein, whey protein, or the leucine metabolite beta-hydroxy-beta-methylbutyrate (HMB), with doses ranging from 6–40 g per day; 20–40 g per day; and 1.5–3 g per day, respectively [39]. The duration of the intervention ranged from 8 weeks to 1 year [39].

However, the meta-analysis of Wand et al. suggests that muscle measures at baseline are predictors of future activities of daily living (ADL) and instrumental activities of daily living (IADL) dependence in the older adult population [40]. Their studies show the association between baseline muscle mass (low vs. high) and muscle strength (handgrip strength, low vs. high) with ADL and IADL at follow-up, as well as the association between SPPB (low vs. high) and gait speed (low vs. high) with ADL and/or IADL at follow-up [40].

A systematic review and meta-analysis by Liao et al. points out that protein supplementation combined with resistance exercise training may have a stronger effect on preventing ageing-related muscle mass attenuation and leg strength loss in older people compared with resistance exercise training alone [41]. Regarding the amount of protein, in most studies included in this systematic review and meta-analysis, protein supplements such as whey protein, leucine, casein, milk protein, and HMB were used (doses ranging from 10 to 35 g/d). The studies show that the participants had substantially greater lean mass and leg strength gains when protein supplementation and resistance exercise training were used than with resistance exercise training alone. The subgroup of studies with a mean BMI ≥ 30 exhibited substantially greater lean mass and leg strength gains in response to protein supplementation. The subgroup of studies with a mean BMI < 30 also exhibited relevant gains in response to protein supplementation [41]. In our study, however, we did not compare the efficacy of whey supplementation with regard to physical activity, although we included studies with such a comparator. Luo et al. indicate that nutritional supplementation may magnify the effect of an exercise intervention on sarcopenia in the elderly [42]. Compared to the exercise group, patients given the dietary supplements had greater increases in lean mass and muscle mass and showed improvements in extension force and normal speed [42]. A systematic review and meta-analysis by Wu et al. show that, compared with the control group, exercise and the combination of exercise and nutrition significantly improved dynamic balance and increased handgrip strength [43].

In a systematic review and meta-analysis by Lighthart-Melis et al. [44], ten studies (2427 participants) showed a high association and considerable overlap (49.7%) between physical (pre-) frailty and (risk of) malnutrition, while seven studies (2506 participants) showed a high association and considerable overlap (41.6%) between sarcopenia and (risk of) malnutrition. The authors suggest that, since the association between the prevalence of (pre-) frailty or sarcopenia and (risk of) malnutrition in older adults is substantial, standardised screening for these conditions is highly warranted to guide targeted nutritional and physical interventions [44].

### 4.3. Strengths and Limitations

As compared to other meta-analyses testing the efficacy of whey protein as a prevention measure towards sarcopenia, the biggest advantage of our systematic review with meta-analysis is meta-regression analysis with the following covariates: study duration (weeks), percentage of males in the population, age of the participants, dose of the active product, percentage of the analysed participants, the changes in appendicular muscle mass rate, handgrip strength, chair and stand test rate, SPPB, and weight by protein intake, although we found no significant result with respect to this data.

Nevertheless, there are some limitations to the present study. The search covered all studies published until 31 December 2022. The most recent studies were not taken into account. The present systematic review and meta-analysis were limited by language criteria (Polish, English, and German), which may have resulted in the exclusion of some studies published in other languages. High variability in terms of classification and cut-off points adopted for the purpose of defining sarcopenia may have had an effect on the heterogeneity of studies and prevented a reliable assessment of the risk of bias. Moreover, according to the adopted criteria, there were only a few studies on the issue, and most of them were in fact database repetitions. Additionally, only ten studies were selected for meta-analysis. All of these were designed as follows: whey protein as an intervention, and other alternatives as a comparator. A placebo and whey intervention would have been an ideal design. Unfortunately, the very low number of studies (only ten) makes it very difficult to ascribe sufficient weight to the conclusions, as one study can easily change the results from significant to non-significant. Thus, the results should be taken into account with caution. However, the correlational analysis, although based on a relatively small number of studies, provides some relationships that could be followed up in subsequent studies.

### 4.4. Implications for Current Practise and Future Research

Sarcopenia is most commonly diagnosed at a critical stage when the patient already experiences severe functional impairment. Therefore, it seems essential to conduct the assessment of the nutritional status of older adults at risk of sarcopenia with an evaluation of body mass and nutritional profile, together with the amount of ingested protein and selected blood parameters, which allow maintaining the correct muscle mass and appropriate physical performance. Interventions such as protein, and vitamin D and E supplementation, as well as interventions aimed at improving physical performance, should be taken, particularly with respect to the group of older adults at risk of sarcopenia.

## 5. Conclusions

The present meta-analysis demonstrated overall that whey supplementation does not improve any of the tested sarcopenia-linked parameters. However, we found that study duration (weeks) and age significantly affect the handgrip strength rate and the chair and stand test rate, respectively, so consideration should be given to oral supplementation combined with the age of participants and an appropriate physical activity as a form of sarcopenia prevention in the high-risk group.

Owing to the moderate quality of evidence and a limited number of studies on the issue, it is warranted to conduct further randomised studies on a larger scale to deepen the understanding of the effect of whey-protein supplementation in combined with age- and need-appropriate exercise for the elderly with sarcopenia.

## Figures and Tables

**Table 1:** Characteristics of the included studies.

No.	Reference (Localisation)	Age [Years] (Mean ± SD)	Subjects/ Males (n)	Health Care Setting	Funding	Intervention	Daily Dose (g)	Ingredients (Names)	Comparator	Duration (Week)	Conclusion
1.	Bauer et al., 2015 [24] (multicentre)	77.71 ± 6.87	380/131	Out patient/ In patient	Industry	Leucine enriched whey protein and vitamin D	2 × 40	WP-MND (FortiFit, Nutricia N.V., The Netherlands)	PBO	13	Whey protein intervention improves muscle mass and lower-extremity function in sarcopenic older adults
2.	Bauer et al., 2020 [25] (multicentre)	77.71 ± 6.87	233/131	Out patient/ In patient	Industry	Leucine enriched whey protein, calcium, and vitamin D	2 × 21	WP-MND (FortiFit, Nutricia N.V., The Netherlands)	PBO	13	No impact of whey protein intervention on kidney function deterioration or symptoms of vitamin D or calcium toxicity
3.	Björkman et al., 2020 [26] (Finland)	83.64 ± 4.9 (P vs. C) 83.8 ± 4.32 (P vs. I)	218/92	Out patients	Academia/ Government	(1) Control with no supplementation (2) Isocaloric placebo (3) Whey protein All groups were given instructions on home-based exercise, dietary protein and vitamin D intake	2 × 20	nd	PBO	52	Whey protein intervention and low-intensity home-based physical exercise did not attenuate the deterioration of muscle and physical performance in sarcopenic older adults
4.	Bo et al., 2019 [27] (China)	74.03 ± 6.29	60/27	nd	Industry/ Academia	Whey protein, vitamin D, and vitamin E	2 × 23	nd	PBO	26	“Whey protein intervention significantly improves RSMI, muscle strength, and anabolic markers such as IGF-I and IL-2 in older adults with sarcopenia”
5.	Hameed, 2018 [28] (Iraq)	77.71 ± 6.87	380/131	nd	nd	Leucine enriched whey protein and vitamin D	2 × 20	nd	PBO	13	Whey protein intervention improves muscle mass and lower-extremity function in sarcopenic older adults
6.	Hill et al., 2019 [29] (multicentre)	77.71 ± 6.87	302/131	Out patient/ In patient	Industry	Leucine enriched whey protein, calcium, and vitamin D	1 × 20	WP-MND (FortiFit, Nutricia N.V., The Netherlands)	PBO	13	“Whey protein intervention improved 25(OH)D, suppressed PTH and had small but positive effects on BMD, indicative of improved bone health, in sarcopenic non-malnourished older adults”
7.	Liberman et al., 2019 [30] (multicentre)	77.72 ± 6.82	288/128	Out patient/In patient	Industry	Leucine enriched whey protein and vitamin D	2 × 20	WP-MND (FortiFit, Nutricia N.V., The Netherlands)	PBO	13	“Whey protein intervention may attenuate the progression of CLIP in older sarcopenic persons with mobility limitations”
8.	Rondanelli et al., 2016 [31] (Italy)	80.51 ± 7.44	130/53	In patients	Industry	Whey protein, amino acids with leucine, and vitamin D Regular controlled physical activity	1 × 22	nd	PBO	12	Whey protein intervention and age-appropriate exercise improve fat-free mass and strength in sarcopenic older adults
9.	Rondanelli et al., 2020 [32] (Italy)	81.5 ± 6.11	127/43	In patients	Industry/ Academia	Leucine enriched whey protein and vitamin D; Rehabilitation	2 × 20	Fortifit^®^, Nutricia	PBO	From 4 to 8	Whey protein intervention improves physical performance, function, and muscle mass in sarcopenic older adults
10.	Verlaan et al., 2018 [33] (multicentre)	77.73 ± 6.88	380/258	Out patient/ In patient	Industry	Leucine enriched whey protein and vitamin D	2 × 20	WP-MND (FortiFit, Nutricia N.V., The Netherlands)	PBO	13	“Whey protein intervention increases muscle mass and improves lower-extremity function in sarcopenic older adults”

nd—no data; source: the authors’ own analysis, PBO—placebo Qualitative analysis.

**Table 2:** Measurement of sarcopenia in the analysed studies.

No.	Reference (Localisation)	(EWGSOP) Criteria (Yes/No)	Definition of Sarcopenia	Body Composition Assessment (DXA/BIA) and Nutritional Status (BMI, MNA)	Muscle Strength and Physical Performance Assessment				
					Handgrip Strength	SPPB	Chair-Stand Time	Balance Test	Gait Speed
1.	Bauer et al., 2015 [24] (multicentre)	YES (EWGSOP)	Sarcopenia was defined as “the age-related loss of muscle mass, strength, and function makes up a large component of physical frailty”	DXA/ BMI by BIA, MNA	Handgrip dynamometry	SPPB score	Chair-stand test score	Balance tests	4-m walk
2.	Bauer et al., 2020 [25] (multicentre)	YES (EWGSOP)	Sarcopenia was defined as “low skeletal muscle mass index (SMI) combined with mild to moderate limitations in physical performance”	BMI, MNA-SF	nd	nd	nd	nd	nd
3.	Björkman et al., 2020 [26] (Finland)	YES (EWGSOP)	Sarcopenia was defined as “low muscle strength, low muscle quantity or quality, and low physical performance”	BIA, tetrapolar BIS device	Handgrip dynamometry	nd	Chair-stand test score	nd	4-m walk
4.	Bo et al., 2019 [27] (China)	YES (EWGSOP)	(1) “the RSMI < 5.7 kg/m^2^ for women and <7.0 kg/m^2^ for men using bioelectric impedance analysis (BIA, Inbody 720); (2) handgrip strength < 18 kg for women and <26 kg for men, or 6-m usual walk speed <0.8 m/s	BIA, MNA	Handgrip dynamometry	nd	Chair stand test score	nd	6-m walk
5.	Hameed, 2018 [28] (Iraq)	YES (EWGSOP)	“Sarcopenia was measured using hydraulic hand dynamometer, SPPB (balance, chair stand test, and gait speed)”	MNA	Handgrip dynamometry	SPPB score	Chair-stand test score	Balance tests	nd
6.	Hill et al., 2019 [29] (multicentre)	nd	“Sarcopenia was determined by Short Physical Performance Battery (SPPB; 0–12) scores between 4 and 9, and a low skeletal muscle mass index (SMI; skeletal muscle mass/BW × 100) ≤ 37% in men and ≤ 28% in women using bioelectric impedance analysis”	DXA, BIA/ BMI, MNA	nd	SPPB score	nd	nd	nd
7.	Liberman et al., 2019 [30] (multicentre)	YES (EWGSOP)	Sarcopenia was defined as “a muscle failure disease that is caused by adverse muscle changes that accumulate over life”	DXA, BMI	nd	SPPB score	nd	nd	nd
8.	Rondanelli et al., 2016 [31] (Italy)	YES (EWGSOP)	Sarcopenia was defined as “the age-related depletion of skeletal muscle mass and loss of strength”	DXA, BIA	Handgrip dynamometry	nd	Chair-stand test score	nd	nd
9.	Rondanelli et al., 2020 [32] (Italy)	YES (EWGSOP)	Sarcopenia was defined “according to European Working Group on Sarcopenia in Older People (EWGSOP) 2010 criteria in terms of the outcome of body composition by bioimpedance analysis [(skeletal muscle mass/body weight × 100) ≤ 37% in men and ≤28% in women], handgrip strength, and gait speed”	DXA, BIA	Handgrip dynamometry	SPPB score	Chair-stand test score	Balance tests	4-m walk
10.	Verlaan et al., 2018 [33] (multicentre)	YES (EWGSOP)	Sarcopenia was defined as “the geriatric syndrome characterized by low muscle mass, strength, and function”	DXA/ BMI by BIA, MNA-SF	Handgrip dynamometry	SPPB score	Chair-stand test score	nd	4-m walk

nd—no data;; source: the authors’ own analysis, EWGSOP: European Working Group for Sarcopenia in Older People.

**Table 3:** Assessment of the risk of bias in the included studies.

Reference (Localisation)	Random Sequence Generation (Selection Bias)	Allocation Concealment (Selection Bias)	Blinding of Participants and Personnel (Performance Bias)	Blinding of Outcome Assessment (Detection Bias)	Incomplete Outcome Data	Selective Reporting (Reporting Bias)	Other Sources of Bias	Number of Low Risk of Bias Assessments	Final Assessment of Study Quality
Bauer et al., 2015 [24]	?	L	L	L	L	L	?	5	HIGH
Bauer et al., 2020 [25]	?	L	L	L	L	L	?	5	HIGH
Björkman et al., 2020 [26]	?	L	L	L	L	L	?	5	HIGH
Bo et al., 2019 [27]	L	L	L	L	L	L	?	6	HIGH
Hameed et al., 2018 [28]	H	H	?	?	H	H	?	0	LOW
Hill et al., 2019 [29]	L	?	L	L	L	L	?	5	HIGH
Liberman et al., 2019 [30]	L	L	?	?	L	L	?	4	HIGH
Rondanelli et al., 2016 [31]	L	L	L	L	L	L	?	5	HIGH
Rondanelli et al., 2020 [32]	L	L	L	L	L	L	?	6	HIGH
Verlaan et al., 2018 [33]	?	?	?	?	L	L	?	2	?

L—low risk of bias; H—high risk of bias; ?—unclassified risk of bias; source: the authors’ own analysis.

**Table 4:** Meta-regression analysis for study duration (weeks), percentage of males in the population, age of the participants (mean), dose of the active product, and percentage of the analysed participants on appendicular muscle mass rate in persons receiving intervention.

Covariates	Appendicular Mass					
	Q	df	Coefficient	SE	Z	*p*
Study duration (weeks)	0.02	1	−0.0022	0.0157	−0.14	0.8868
% male	0.02	1	0.0039	0.0255	0.15	0.8773
Age	0.10	1	0.0123	0.0396	0.31	0.7566
Dose	0.02	1	−0.0005	0.0034	−0.15	0.8769
% analysed	0.37	1	−0.0004	0.0006	−0.61	0.5443
	**Handgrip strength**					
	**Q**	**df**	**Coefficient**	**SE**	**Z**	***p***
Study duration (weeks)	5.01	1	−0.0083	0.0037	−2.24	0.0252
% male	2.76	1	0.0407	0.0245	1.66	0.0968
Age	0.47	1	−0.0311	0.0455	−0.68	0.4934
Dose	0.84	1	−0.0051	0.0056	−0.92	0.3589
% analysed	3.14	1	−0.0018	0.0010	−1.77	0.0765
	**Chair and stand test**					
Study duration (weeks)	29.12	1	−0.1522	0.0282	−5.40	0.0000
% male	2.72	1	−0.8611	0.5221	−1.65	0.0991
Age	29.20	1	0.2822	0.0522	5.40	0.0000
Dose	0.09	1	−0.0031	0.0104	−0.30	0.7625
% analysed	29.12	1	−0.0042	0.0008	−5.40	0.0000
	**SPPB**					
Study duration (weeks)	0.21	1	−0.0012	0.0027	−0.45	0.6503
% male	0.00	1	−0.0010	0.0291	−0.03	0.9739
Age	0.04	1	0.0039	0.0187	0.21	0.8353
Dose	0.01	1	−0.0002	0.0025	−0.07	0.9415
% analysed	0.72	1	−0.0005	0.0006	−0.85	0.3971
	**Weight**					
Study duration (weeks)	1.96	1	−0.0048	0.0034	−1.40	0.1615
% male	0.03	1	0.0027	0.0145	0.18	0.8537
Age	0.00	1	0.0015	0.0263	0.06	0.9557
Dose	0.10	1	−0.0029	0.0093	−0.31	0.7571
% analysed	0.41	1	−0.0008	0.0013	−0.64	0.5232
